# Adult Body Height and Cardiometabolic Disease Risk: The China National Health Survey in Shaanxi

**DOI:** 10.3389/fendo.2020.587616

**Published:** 2020-12-21

**Authors:** Yuan Yuan, Bo Zhou, Shunan Wang, Jia Ma, Fen Dong, Min Yang, Zhixin Zhang, Wenquan Niu

**Affiliations:** ^1^Graduate School, Beijing University of Chinese Medicine, Beijing, China; ^2^International Medical Services, China-Japan Friendship Hospital, Beijing, China; ^3^Department of Pediatrics, Oriental Hospital Affiliated to Beijing University of Chinese Medicine, Beijing, China; ^4^Institute of Clinical Medical Sciences, China-Japan Friendship Hospital, Beijing, China

**Keywords:** body height, cardiometabolic disease, adult, optimal model, risk prediction

## Abstract

**Objectives:**

Based on data from the China National Health Survey, we aimed to examine the association between body height and cardiometabolic disease (CMD) in a large adult population from Shaanxi province, and further to test whether this association was hinged upon other population characteristics.

**Methods:**

This population-based study was conducted in 2014 in Shaanxi Province, China. Utilizing a multi-stage stratified cluster sampling method, total 5,905 adults with complete data were eligible for analysis, and 1,151 (19.5%) of them had CMD. Of 1,151 CMD patients, 895 (15.1%) had one disorder and 256 (4.4%) had ≥2 disorders.

**Results:**

Using the bi-directional stepwise method and all-subsets regression, five factors—age, body mass index, family histories of CMD, exercise, and height—constituted the optimal model when predicting CMD risk. Restricted cubic spline regression showed a reduced tendency towards CMD with the increase of body height, with per 10 cm increment in body height corresponding to 14% reduced risk. Ordinal Logistic regression supported the contribution of body height on both continuous and categorical scales to CMD risk before and after adjustment, yet this contribution was significantly confounded by exercise and education, especially by exercise, which can explain 65.4% of total impact. For example, short stature was associated with an increased risk of CMD after multivariable adjustment not including exercise and education (odds ratio, 95% confidence interval, P: 1.42, 1.21 to 1.66, <0.001), and tall stature was associated with a reduced risk (0.77, 0.64 to 0.92, 0.003).

**Conclusions:**

Our findings indicate short stature was a risk factor, yet tall stature was a protective factor for CMD in Chinese. Notably, the prediction of short and tall stature for CMD may be mediate in part by exercise.

## Introduction

Cardiometabolic disease (CMD) includes a constellation of cardiac and metabolic disorders with shared pathogeneses or interlinked mechanisms, and some components of CMD, such as hypertension and type 2 diabetes have posed major public health burdens around the globe (1, 2). Currently, much attention has been paid to CMD, due to its resultant adverse clinical consequences (3, 4). Hence, unraveling the risk profiles of CMD and managing it from a holistic point of view remains a challengeable task.

As CMD develops through a multistep and multifactorial process, it is of great interest to identify widely available and easily identified characteristics that can help predict the risk of CMD, such as body height. There is evidence indicating that short statured persons were observed to be more likely to have abnormal blood pressure (5), unfavorable glucose and lipid metabolisms (6, 7), as well as cardiovascular and cerebrovascular events (8, 9). In support of this claim, an individual participant meta-analysis conducted by the Emerging Risk Factors Collaboration showed that taller adults had a lower risk of deaths from coronary disease, stroke subtypes, and heart failure than shorter adults (10). By contrast, other studies reported a reduced risk of death rates from CMD among short statured persons (11). Existing data on the relationship between body height and CMD are conflicting and are limited almost exclusively to populations of American and European origins. Given the wide geographic and race-specific distributions of adult height worldwide, it is necessary to justify the risk of CMD conferred by body height in specific populations.

To shed some light and based on the data from the China National Health Survey, we aimed to examine the association of body height with CMD risk in a large population from Shaanxi province, China, and further to test whether this association was hinged upon other population characteristics.

## Methods

### Ethical Approval and Informed Consent

The conduct of this survey was reviewed and approved by the Institute of Basic Medical Sciences, Chinese Academy of Medical Sciences. All participants enrolled in this study gave informed consent.

### Study Population

The present study was based on the data collected in 2014 from the China National Health Survey, a population-based cross-sectional survey of Chinese individuals, and the sampling procedure was previously described (12). Briefly, utilizing a multi-stage stratified cluster sampling method, participants who lived in the selected areas in Shaanxi province were invited to complete a detailed questionnaire and physical examination. Finally, 5,905 adult participants with eligible information were enrolled in the final analysis.

### Data Collection

Data on demographics and lifestyles, anthropometric measurements, personal and family medical histories were collected, including age, sex, body height, weight, age at menarche and menopause (only for females), ethnicity, areas, marital status, education, income, smoking status, drinking status, physical activity, exercise, personal and family medical histories of CMD.

### Definitions of CMD and Characteristics

CMD is diagnosed when a person has one or more cardiac and metabolic disorders, including hypertension, type 2 diabetes, hyperlipemia, stroke, and cardiovascular disease. In this study, participants who were free of CMD formed the control group, and CMD patients were classified into two case groups based on the number of above disorders carried (13).

Medical histories were based on diagnosis certificates from second-class or above hospitals. Body height and weight were measured to the nearest 0.1 cm and 0.1 kg, respectively. Body mass index (BMI) was calculated as weight in kilograms divided by the square of body height in meters. Overweight and obesity were defined as BMI ranging from 24 to 27.9 kg/m^2^ and BMI ≥28 kg/m^2^ under the China criteria, respectively (14).

At present, there are no unified standards for short stature in adults, and some previous studies have adopted the lowest quartile or quintile of study population as the upper cutoffs to define short stature (8, 15). In this study, we classified study participants with body height <25^th^ quartile of sex-specific values as short stature, with body height >75^th^ quartile of sex-specific values as tall stature, and with the interval height values as normal stature.

Ethnicity was classified into two categories (Han ethnicity *vs.* other ethnicities). Areas comprised rural and urban areas. Marital status included three categories: single, married, and divorced or widowed. Education was classified into primary school degree or below, secondary degree, high school degree, and college degree or above. Personal income (RMB per month) was stratified into <2,000, 2,000 to 4,000, and >4000. Smoking or drinking status was defined as current or former consumption and never. Physical activity was classified into light, moderate, and heavy activities. Exercise referred to leisure time exercise of at least 20 min each time, and was stratified into never, <1, 1 to 2, 3 to 4, and 5 to 7 times per week.

### Statistical Analyses

Categorical variables are presented as count (percentage). Continuous variables are tested for normality by using the skewness and kurtosis test, and are presented as mean (standard deviation) and median (interquartile range) for normally distributed and skewed variables, respectively. Kruskal-Wallis rank-sum test, t test, and χ^2^ test were used to compare baseline characteristics across normal controls, CMD patients with 1 disorder, and CMD patients with ≥2 disorders, where appropriate.

First, bi-directional stepwise method and all-subsets regression were employed to select contributing factors and determine the optimal model according to root mean square error (RMSE), mean square error (MAE), and R^2^, which were calculated by the “MASS” and “leaps” packages in the R programming environment (version 4.0.2). The related analysis scripts are shown in **Supplementary File 1**. Next, ordinal Logistic regression analyses were performed to examine the crude and adjusted association of body height on both continuous and categorical scales with CMD risk. Two adjusted models were created, with model 1 controlling for age, sex, marital status, personal income, drinking, and family histories of CMD, and model 2 additionally controlling for exercise and education. All controlled factors were modelled as independent terms and in original data forms. Effect-size estimates in ordinal Logistic regression analyses are summarized as odds ratios (ORs) and 95% confidence intervals (95% CIs). Additionally, restricted cubic splines were performed to illustrate the risk distribution of CMD across whole height interval. Based on the results of ordinal Logistic regression analyses, subgroup analyses, and mediation analyses were further implemented, targeting significant confounding factors attributing to height prediction. Statistical power to detect significance was estimated using the PS Power and Sample Size Calculations software version 3.0.

Based on the contributing factors in the optimal model, a wide range of diagnostic statistics were calculated to examine the prediction performance of this optimal model in predicting CMD from both calibration and discrimination aspects. In detail, Akaike information criterion (AIC), Bayesian information criterion (BIC), and the -2-log likelihood ratio test were used to evaluate the calibration. Integrated discrimination improvement (IDI) and area under the receiver operating characteristic (AUROC) were used to evaluate the discrimination.

Finally, a prediction nomogram model for predicting the risk of CMD was created, and calibration curve was drawn to assess prediction accuracy of this model. The nomogram and calibration curve were depicted by using the “rms” package in the R programming environment (version 4.0.2) (16). The related analysis scripts are shown in **Supplementary File 2**.

Statistical analyses were done using the STATA software (version 14.0, Stata Corp, TX, USA) unless otherwise indicated. Two-sided P value less than 0.05 was considered statistically significant. The stringent Bonferroni correction method was adopted in case of multiple comparisons.

## Results

### Baseline Characteristics

The baseline characteristics of 5,905 adults in this study are shown in **Table 1**. A total of 1,151 (19.5%) adults suffered CMD, and there were 895 (15.1%) patients with one disorder and 256 (4.4%) patients with ≥2 disorders.

**Table 1:** The baseline characteristics of all study adults (n = 5,905).

Characteristics	Normal controls	CMD patients with 1 disorder	CMD patients with ≥2 disorders	P
	(n = 4,754)	(n = 895)	(n = 256)	
Age (years)	45 (33, 54)	58 (52, 64)	62.0 (56, 69.5)	0.001
Males	1,927 (40.5%)	392 (43.8%)	119 (46.5%)	0.043
Height (cm)	161 (155.8, 167.7)	160.5 (154.5, 166.8)	160.5 (154.3, 166.5)	0.004
Stature category				
Tall stature (height > highest quartile)	1,267 (26.8%)	178 (20%)	48 (18.8%)	<0.001
Normal stature	2,366 (50%)	434 (48.7%)	117 (45.7%)	
Short stature (height < lowest quartile)	1,100 (23.2%)	279 (31.3%)	91 (35.5%)	
BMI (kg/m^2^)	22.8 (20.7, 25.2)	62.5 (56, 69.5)	25.5 (23.3, 27.6)	0.001
BMI category				
Normal	2,980 (63.2%)	335 (37.8%)	84 (33.2%)	<0.001
Overweight	1,351 (28.6%)	401 (45.2%)	115 (45.5%)	
Obesity	385 (8.2%)	151 (17%)	54 (21.3%)	
Age at menarche (years)				
<13	401 (16.7%)	47 (11.9%)	12 (10.5%)	<0.001
13 to 14	561 (23.3%)	58 (14.7%)	11 (9.6%)	
15 to 16	1,061 (44.1%)	157 (39.8%)	45 (39.5%)	
>16	381 (15.8%)	132 (33.5%)	46 (40.4%)	
Age at menopause (years)				
<45	85 (9.4%)	37 (9.4%)	15 (12.1%)	0.005
45 to 49	393 (43.2%)	138 (34.9%)	44 (35.5%)	
50 to 54	380 (41.8%)	176 (44.6%)	55 (44.4%)	
>54	51 (5.6%)	44 (11.1%)	10 (8.1%)	
Ethnicity				
Han	4,733 (99.6%)	888 (99.2%)	256 (100%)	0.210
Other ethnicities	21 (0.4%)	7 (0.8%)	0 (0%)	
Areas				
Rural	2,357 (49.6%)	465 (52%)	108 (42.2%)	0.022
Urban	2,396 (50.4%)	430 (48%)	148 (57.8%)	
Marital status				
Single	524 (11%)	4 (0.4%)	1 (0.4%)	<0.001
Married	4,104 (86.3%)	822 (91.8%)	224 (87.5%)	
Divorced or widowed	125 (2.6%)	69 (7.7%)	31 (12.1%)	
Education				
Primary school degree or below	580 (12.2%)	264 (29.5%)	92 (35.9%)	<0.001
Secondary school degree	1,665 (35%)	299 (33.4%)	71 (27.7%)	
High school degree	1,139 (24%)	191 (21.3%)	44 (17.2%)	
College degree or above	1,369 (28.8%)	141 (15.8%)	49 (19.1%)	
Personal income (RMB per month)				
<2000	1,915 (40.3%)	453 (50.8%)	109 (42.7%)	<0.001
2000 to 4000	1,729 (36.4%)	247 (27.7%)	86 (33.7%)	
>4000	1,103 (23.2%)	191 (21.4%)	60 (23.5%)	
Smoking				
No	3,421 (72%)	630 (70.4%)	188 (73.4%)	0.528
Yes	1,333 (28%)	265 (29.6%)	68 (26.6%)	
Drinking				
No	4,358 (91.7%)	808 (90.3%)	218 (85.2%)	0.001
Yes	396 (8.3%)	87 (9.7%)	38 (14.8%)	
Physical activity				
Light	3,485 (73.3%)	671 (75%)	212 (82.8%)	0.006
Moderate	556 (11.7%)	88 (9.8%)	15 (5.9%)	
Heavy	713 (15%)	136 (15.2%)	29 (11.3%)	
Exercise (times per week)				
Never	1,364 (28.7%)	383 (42.8%)	146 (57%)	<0.001
<1	611 (12.9%)	122 (13.6%)	26 (10.2%)	
1 to 2	887 (18.7%)	110 (12.3%)	21 (8.2%)	
3 to 4	426 (9%)	61 (6.8%)	14 (5.5%)	
5 to 7	1,461 (30.8%)	218 (24.4%)	49 (19.1%)	
Family histories of CMD				
Category 1 (1 family member)	2,829 (59.5%)	400 (44.7%)	111 (43.4%)	<0.001
Category 2 (2 family members)	1,524 (32.1%)	415 (46.4%)	114 (44.5%)	
Category 3 (3 family members)	399 (8.4%)	80 (8.9%)	31 (12.1%)	

SD, standard deviation; BMI, body mass index; CMD, cardiometabolic disease. Data are expressed as median (interquartile range) for continuous variables or count (percent) for categorical variables. P value was calculated by the t-test, Kruskal-Wallis rank-sum test, or the χ^2^ test, where appropriate.

### Identifying Contributing Factors and Determining the Optimal Model for CMD

As shown in **Table 2**, using the bi-directional stepwise method and all-subsets regression, five factors, including height and other four confounding factors—age, BMI, family histories of CMD, and exercise—were found to be in significant association with CMD and they formed the optimal model, as this model had the lowest PMSE and MAE and the highest R^2^.

**Table 2:** Identification of the best model in predicting CMD by AIC in a stepwise algorithm.

Number*: characteristics	RMSE	MAE	R^2^
1: age	0.4743	0.3438	0.1621
2: age, BMI	0.4690	0.3457	0.1807
3: age, BMI, family histories of CMD	0.4635	0.3443	0.2006
4: age, BMI, family histories of CMD, exercise	0.4629	0.3433	0.2025
5: age, BMI, family histories of CMD, exercise, height	0.4626	0.3431	0.2031
6: age, BMI, family histories of CMD, exercise, height, education	0.4629	0.3432	0.2026

AIC, Akaike information criterion; BMI, body mass index; CMD, cardiometabolic disease; RMSE, root mean square error; MAE, mean square error. *The number of characteristics under the present study in each optimal model.

### Prediction of Body Height for CMD

To further interrogate the risk prediction of body height for CMD, the ordinal Logistic regression analyses were used before and after adjusting for confounding factors (**Table 3**). Body height was analyzed on both continuous and categorical scales. Per 10 cm increment in body height was associated with 14% reduced risk of CMD without adjustment (Model 0) (OR = 0.86, 95% CI: 0.80 to 0.93, P < 0.001), while this risk was reduced by 36% after adjusting for age, sex, marital status, personal income, drinking, and family histories of CMD (Model 1) (OR = 0.64, 95% CI: 0.57 to 0.73, P < 0.001). Further additional adjustment for exercise and education (Model 2) yielded no statistical significance.

**Table 3:** Prediction of body height on both continuous and categorical scales for CMD by using the ordinal Logistic regression analyses.

Body height	OR, 95% CI, P		
	Model 0	Model 1	Model 2
Height (per 10 cm increment)	0.86, 0.80 to 0.93, <0.001	0.64, 0.57 to 0.73, <0.001	0.94, 0.82 to 1.07, 0.340
Height category			
Normal stature	Reference group	Reference group	Reference group
Short stature	1.45, 1.25 to 1.69, <0.001	1.42, 1.21 to 1.66, <0.001	1.12, 0.95 to 1.32, 0.178
Tall stature	0.77, 0.65 to 0.91, 0.002	0.77, 0.64 to 0.92, 0.003	1.02, 0.84 to 1.23, 0.843

CMD, cardiometabolic disease; OR, odds ratio; 95% CI, 95% confidence interval. Under model 0, no confounder was adjusted. Under model 1, age, sex, marital status, personal income, drinking, and family histories of CMD were adjusted, and exercise and education were additionally adjusted under model 2.

Relative to normal stature, short stature was associated with a statistically increased risk of having CMD in Model 0 (OR = 1.45, 95% CI: 1.25 to 1.69, P < 0.001) and Model 1 (OR = 1.42, 95% CI: 1.21 to 1.66, P < 0.001), and similarly no significance was observed in Model 2. Tall stature was associated with a statistically reduced risk of CMD in Model 0 (OR = 0.77, 95% CI: 0.65 to 0.91, P = 0.002) and Model 1 (OR = 0.77, 95% CI: 0.64 to 0.92, P = 0.003), while there was no observable significance in Model 2.

The significance persisted even after adopting the stringent Bonferroni correction (P < 0.05/3 for continuous height and <0.05/6 for categorical height).

**Figure 1:**
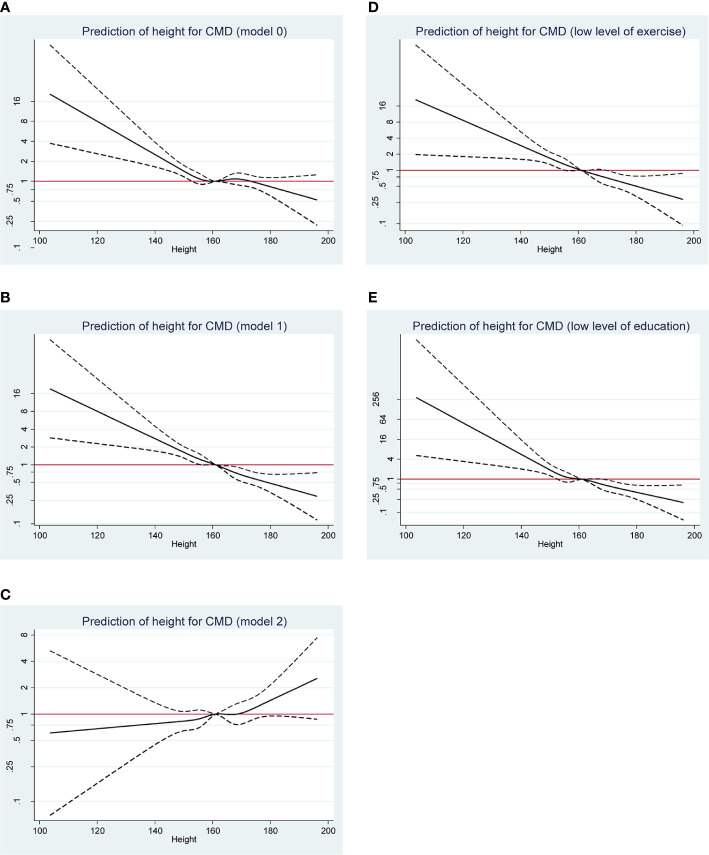
Restricted cubic spline regression analysis on the prediction of body height on a continuous scale for overall CMD risk under the model 0 **(A)**, model 1 **(B)**, and model 2 **(C)**, respectively. Further analyses targeting participants with ≤3 times of exercise per week **(D)** or with education of secondary school degree or below **(E)**. CMD, cardiometabolic disease. Under the model 0, no confounder was adjusted. Under the model 1, age, sex, marital status, personal income, drinking, and family histories of CMD were adjusted, and exercise and education were additionally adjusted under the model 2.

**Figure 1** displays the risk prediction of body height for CMD and its subtypes under the restricted cubic spline regression model. Overall, there was a reduced tendency towards CMD with the increase of body height when combining CMD patients as a whole without adjustment (Model 0) (panel A), as well as with partial adjustment (Model 1) (panel B). Without observable significance in Model 2, an opposite tendency was observed (panel C). However, further analyses targeting participants with ≤3 times of exercise per week or with education of secondary school degree or below showed the apparent reduced tendency (panels D and E).

### Impact of Exercise and Education on the Height-CMD Association

In view of the confounding impact of exercise and education on the risk prediction of body height for CMD, subgroup analyses were undertaken according to the two confounders respectively (**Table 4**). Significance was only noted in subgroups with ≤3 times of exercise per week and with education of secondary school degree or below. The estimated power to detect significance in subgroup analyses were over 85%.

**Table 4:** Prediction of body height on both continuous and categorical scales for CMD stratified by exercise and education, respectively.

Characteristics	OR, 95% CI, P*		
	Height (per 10 cm increment)	Short stature *vs.* Normal stature	Tall stature *vs.* Normal stature
**Exercise (times per week)**			
>3	0.82, 0.65 to 1.04, 0.103	1.09, 0.97 to 1.23, 0.142	0.78, 0.56 to 1.08, 0.128
≤3	0.66, 0.47 to 0.93, 0.017	1.29, 1.13 to 1.47, <0.001	0.75, 0.61 to 0.93, <0.001
**Education**			
High school degree or above	1.11, 0.92 to 1.34, 0.294	0.97, 0.84 to 1.12, 0.660	1.02, 0.80 to 1.30, 0.854
Secondary school degree or below	0.84, 0.73 to 0.97, 0.015	1.22, 1.09 to 1.37, <0.001	0.65, 0.50 to 0.85, <0.001

CMD, cardiometabolic disease; OR, odds ratio; 95% CI, 95% confidence interval. ^*^P was calculated after adjusting for age, sex, marital status, personal income, drinking, and family histories of CMD.

Further mediation test (**Supplementary File 3** and **Supplementary Figure 1**) indicated that exercise was a significant mediator to the extent to which it carried the significant impact of body height on a continuous scale on CMD risk, and the proportion of total impact conferred by exercise was estimated to be 65.4%. By contrast, the proportion of total effect conferred by education was estimated to be 24.6%.

### Model Diagnostics

**Figure 2:**
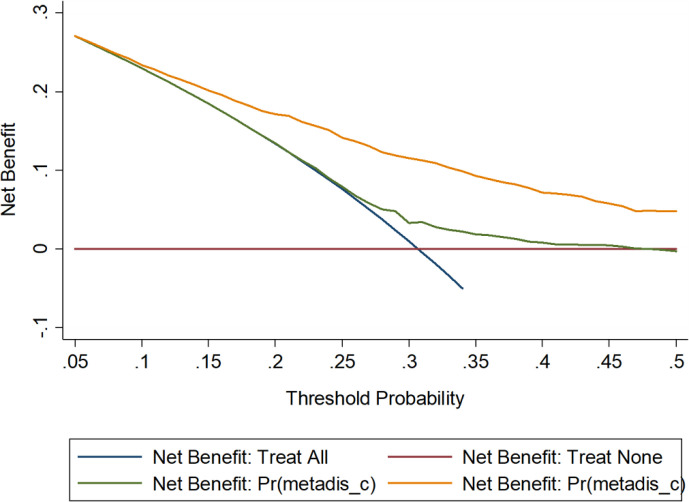
Decision curve analysis on the net benefits gained by the five factors in the optimal model when predicting CMD risk. CMD, cardiometabolic disease. The orange solid line corresponds to the basic model that includes sex, ethnicity, area, marital status, personal income, physical activity, smoking, and drinking. The green solid line corresponds to the full model that includes all factors in the basic model and the five factors in the optimal model including age, body mass index, family histories of CMD, exercise, and height.

To facilitate the practical use of the optimal model (including five factors) determined aforementioned, model diagnostics were assessed by a wide range of statistics from calibration and discrimination aspects (**Table 5**), and by a visual inspection in decision curve analysis (**Figure 2**). Overall, the prediction performance was obviously improved by adding the factors in the optimal model to the basic model (including sex, ethnicity, area, marital status, personal income, physical activity, smoking, and drinking).

**Table 5:** Prediction performance assessment of five optimal characteristics for CMD in all study adults.

Statistics	Basic model	Full model
**Calibration**		
AIC	4,221.38	3,636.53
BIC	4,276.73	3,722.46
LR test (χ^2^)	552.15	
LR test (P value)	<0.001	
**Discrimination**		
IDI (P value)	<0.001	
AUROC (P value)	<0.001	

CMD, cardiometabolic disease; AIC, Akaike information criterion; BIC, Bayesian information criterion; LR, likelihood ratio; AUROC, the area under the receiver operating characteristic. Basic model included sex, ethnicity, area, marital status, personal income, physical activity, smoking, and drinking, and full model additionally included five optimal characteristics, including age, body mass index, family histories of CMD, exercise, and height.

### Prediction Nomogram Model

**Figure 3:**
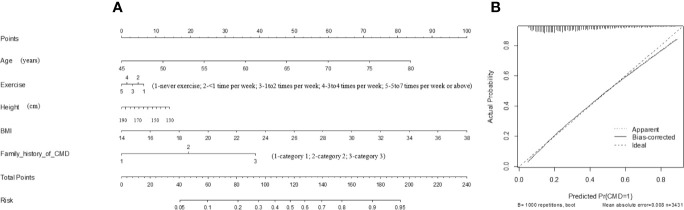
The prediction nomogram model of the five factors in the optimal model for quantifying the risk of CMD **(A)**, as well as the calibration curve of this model **(B)**. CMD, cardiometabolic disease; BMI, body mass index. This nomogram can be used to manually obtain predicted values from a regression model that was fitted with the five factors. In detail, there is a reference line at the top for reading scoring points (range: 0 to 100) from all factors in the regression model, which were summed together to calculate the total points, and then the predicted values can be read at the bottom.

A prediction nomogram model based on factors in the optimal model was finally generated in predicting the risk of CMD (**Figure 3**). The prediction accuracy of this model was good, with the C-index of being 74.4%.

Assuming an adult aged 62 years old (40 points), doing two times of exercise per week (3 points), with body height of 170 cm (6 points), with BMI of 32 kg/m^2^ (75 points), having two family members diagnosed with CMD (20 points), the total point was summed as 144, and the risk of having CMD was estimated to be 75%.

## Discussion

In this large, population-based analysis on 5,905 Chinese adults from Shaanxi province, we aimed to examine the association of body height with CMD, and further to test whether this association was hinged upon other population characteristics. The key finding of this study is that the risk of CMD was reduced with the increase of body height, that is, short stature was a risk factor, yet tall stature was a protective factor. Moreover, the prediction of short and tall stature for CMD was confounded by exercise and education, and especially over two thirds of the prediction can be explained by exercise. To our knowledge, this is the pilot study that has investigated the impact of body height on CMD among Chinese adults.

Divergent association between body height and health outcomes have been reported in the medical literature. Our findings are in line with that of some previous studies by showing that short stature was a risk predictor for CMD (17, 18), yet the underlying reasons remain largely unknown. It is possible that the relationship between adult height and CMD can be, at least in part, explained by genetic factors (19). From organ function point of view, short statured persons tend to have a worse lung function (18), which can precipitate the development of subsequent CMD. Besides, adult height is widely regarded as a marker of early-life exposures, such as nutrition and diseases (20), which indirectly contribute to the risk of having CMD. Also, there is evidence that adult height can be influenced by hormonal factors. For example, thyroid hormone, playing a decisive role in height gain, are linked with cardiometabolic progress (21). Therefore, it is reasonable to speculate that short statured persons are more likely to suffer CMD.

It is worth noting that our results further revealed the impact of body height on CMD was confounded by exercise and education. Exercise is known as a natural strong metabolism-improving strategy (22), and it is widely applied in management of cardiovascular diseases and diabetes. In addition, well-designed exercise increases growth hormone levels, and is beneficial for organic metabolism (23). On the other hand, there is also a close association between exercise and body height (24). We accordingly did a mediation test in our study, showing that near two thirds of the impact of body height on CMD was conferred by exercise, indicating that concerns over exercise should be proposed among adults with short stature.

Similarly, education is regarded as an advantageous factor that is inversely associated with CMD (25). People with high education tend to have a high level of health literacy, leading to health behavior and health status (26). And education is also interacted with body height (27), accounting for less than one forth impact of body height on CMD in mediation test. Thus, there is an urgent need to raise health literacy in adults with short stature.

To facilitate the practical application of our findings, on the basis of the optimal model determined by the bi-directional stepwise method and all-subsets regression, we constructed a prediction nomogram model after multi-angle model diagnostic checking, and this model had a good prediction accuracy. We believed, when confirmed, our findings may have implications for risk stratification and the management of CMD in Chinese.

### Strengths and Limitations

Strengths of this study include a comprehensive analysis of the association between body height and CMD risk in a large-scale Chinese population. Additionally, our study provides accordance on making more efficient and more effective preventive strategies for CMD.

Some limitations should be acknowledged when interpreting our findings. Firstly, this is a cross-sectional study, which precludes further comments on causality. For example, body height can only be regarded as an indicator in this study, rather than a determinant of CMD, calling for further longitudinal studies to confirm or refute our conclusions. Secondly, this study was based on adults living in Shaanxi, China, and so the results may not generalizable to the other populations. Thirdly, adult height in this study was recorded only once at the time of enrollment, and it is of added interest to examine the changes in height, especially females, who will become shorter with aging.

### Conclusions

Taken together, our findings indicate short stature was a risk factor, yet tall stature was a protective factor for CMD in Chinese. Notably, the prediction of short and tall stature for CMD was confounded by exercise and education, and especially over two thirds of the prediction can be explained by exercise. For practical reasons, we hope this study will not represent just an endpoint of investigation instead of a start to establish background data to further explore the correlation between body height and the development of CMD, as well as the underlying mechanisms in the future.

## Data Availability Statement

The raw data supporting the conclusions of this article will be made available by the authors, without undue reservation.

## Ethics Statement

The studies involving human participants were reviewed and approved by the Institute of Basic Medical Sciences, Chinese Academy of Medical Sciences. The patients/participants provided their written informed consent to participate in this study.

## Author Contributions

ZZ planned and designed the study and directed its implementation. ZZ drafted the protocol. YY, BZ, JM, and ZZ obtained statutory and ethics approvals. YY, SW, MY, and ZZ contributed to data acquisition. YY and WN conducted statistical analyses. YY, BZ, FD, and WN did the data preparation and quality control. YY and WN wrote the manuscript. All authors contributed to the article and approved the submitted version.

## Conflict of Interest

The authors declare that the research was conducted in the absence of any commercial or financial relationships that could be construed as a potential conflict of interest.
