# Supplementary material for: Adult Body Height and Cardiometabolic Disease Risk: The China National Health Survey in Shaanxi
Source: Front Endocrinol (Lausanne). 2020 Dec 21;11:587616. doi: 10.3389/fendo.2020.587616 (PMC7780292; doi:10.3389/fendo.2020.587616)
Supplement: Supplementary file 1 [file Table_1.docx]

**Supplementary File 1. The analysis scripts for identifying significant factors associated with cardiometabolic disease using the stepwise method and all-subsets regression in the R programming environment (version 4.0.2).**

dc<-read.table("data.txt", head=T)

attach(dc)

library(MASS)

full.model <- glm(metadis ~ age + sex + height + bmi + meno + me + race + nowplace + marital + edu + income + smoke + drink + activities + exercise + famdis, family=binomial(), data=dc)

step.model <- stepAIC(full.model, direction = "both")

step.model$anova

summary(step.model)

library(leaps)

models <- regsubsets(metadis ~ age + height + bmi + edu + exercise + famdis, data = dc, nvmax = 6, method = "seqrep")

summary(models)

dc<-read.table("data2.txt", head=T)

attach(dc)

set.seed(2020)

library(caret)

train.control <- trainControl(method = "cv", number = 10)

step.model <- train(metadis ~ age + sex + height + bmi + edu + exercise + famdis, data = dc, na.action=na.delete, method = "leapBackward", tuneGrid = data.frame(nvmax = 1:6), trControl = train.control )

step.model$results

**Supplementary File 2. The analysis scripts for the nomogram plot and calibration curve using the “rms” package in the R programming environment (version 4.0.2).**

require(rms)

dc<-read.table("data.txt", head=TRUE)

rr<-datadist(dc)

options(datadist="rr")

f <- lrm(CMD~ Age + Height + Exercise + BMI_category + Family_history_of_CMD, data=dc, x=TRUE, y=TRUE)

nom <- nomogram(f, fun=plogis, fun.at=c(0.001, 0.01, 0.05, seq(0.1, 0.9, by=0.1), 0.95, 0.99, 0.999), lp=F, funlabel="Risk")

plot(nom)

cal <- calibrate(f, method="boot", u=120, m=220, B=1000)

plot(cal)

rcorrcens(CMD ~ predict(f), data =dc)

**Supplementary File 3. The analysis scripts for the mediation analysis on the impact of two significant characteristics on the association between body height and cardiometabolic disease in the R programming environment (version 4.0.2).**

dc<-read.table("data.txt", head=TRUE)

library(mediation)

attach(dc)

b <- lm(Exercise~ height + Age + Education+ BMI_category + Family_history_of_CMD, data=dc)

c <- lm(CMD~ height + Age + Education + Exercise +BMI_category + Family_history_of_CMD, data=dc)

contcont <- mediate(b, c, sims=2000, treat="height", mediator="Exercise")

summary(contcont)

plot(contcont)

d <- lm(Education~ height + Age + Exercise + BMI_category + Family_history_of_CMD, data=dc)

e <- lm(CMD~ height + Age + Education + Exercise +BMI_category + Family_history_of_CMD, data=dc)

contcont <- mediate(d,e sims=2000, treat="height", mediator="Education")

summary(contcont)

plot(contcont)

**Supplementary Figure 1. The mediation effect of exercise (panel A) and education (panel B) on the association between body height and cardiometabolic disease.**

B

A

Abbreviations: ACME: average causal mediation effect; ADE: average direct effect.
